# Multi-Marker Approach in Sepsis: A Clinical Role Beyond SOFA Score

**DOI:** 10.3390/medicina62010201

**Published:** 2026-01-18

**Authors:** Gun Hyuk Lee, Hanah Kim, Hee-Won Moon, Yeo-Min Yun, Seungho Lee, Mina Hur

**Affiliations:** 1 Department of Laboratory Medicine and Genetics, Samsung Medical Center, Sungkyunkwan University School of Medicine, Seoul 06351, Republic of Korea; leegunhyuk93@gmail.com; 2 Department of Laboratory Medicine, Konkuk University School of Medicine, Seoul 05030, Republic of Korea; md.hkim@gmail.com (H.K.); hannasis@hanmail.net (H.-W.M.); yun7640@gmail.com (Y.-M.Y.); 3 Department of Preventive Medicine, College of Medicine, Dong-A University, Busan 49201, Republic of Korea; lgydr1@gmail.com

**Keywords:** sepsis, SOFA score, procalcitonin, presepsin, interferon, adrenomedullin

## Abstract

*Background and Objectives*: Procalcitonin (PCT), presepsin (PSEP), interferon-λ3 (IFN-λ3), and bioactive adrenomedullin (bio-ADM) are promising sepsis biomarkers. We explored the clinical utility of a multi-marker approach using these four biomarkers in patients with suspected sepsis. *Materials and Methods*: In a total of 248 patients, the biomarkers were evaluated with the sequential organ failure assessment (SOFA) score. Receiver operating characteristic curves with area under the curve (AUC) were analyzed to diagnose sepsis and predict in-hospital mortality. Survival and reclassification analyses were also used to predict in-hospital mortality. *Results*: The four biomarkers showed comparable diagnostic performance (AUC = 0.61–0.95, *p* < 0.001–0.003), and sepsis proportion increased significantly as the number of biomarkers used in the multi-marker approach increased (7.7–91.7%, *p* < 0.001). The proportion of biomarker quartiles (Q1–Q4) differed significantly according to SOFA score (*p* < 0.001). The four biomarkers predicted in-hospital mortality (AUC = 0.63–0.84, *p* < 0.001–0.004). The multi-marker approach performed better than the SOFA score (mortality rate, 58.3% vs. 31.3%; adjusted hazard ratio [HR], 14.7 vs. 4.6), and the addition of biomarkers to the SOFA score increased the performance. The multi-marker approach resulted in a higher HR in patients aged ≥75 years than in the overall population (9.2 vs. 4.2). *Conclusions*: Each biomarker showed clinical utility in patients with suspected sepsis. The multi-marker approach showed complementary clinical utility in addition to the SOFA score and better prognostic performance in patients aged ≥75 years. The use of biomarkers, alone or in combination, would be a valuable tool in combination with the SOFA score.

## 1. Introduction

In 2017, an estimated 48.9 million cases of sepsis (95% uncertainty interval, 38.9–62.9) and 11.0 million sepsis-related deaths (10.1–12.0) were reported globally [1]. The average 30-day mortality rate for sepsis was 24.4% in a meta-analysis, reflecting the persistently high fatality associated with sepsis despite advances in critical care [2]. According to the CDC data, the sepsis-related death rate in adults aged ≥85 years (750.0 per 100,000) was approximately five times higher than that in those aged 65–74 years (150.7 per 100,000) [3]; in critically ill patients, an age ≥75 years has been identified as an independent risk factor for mortality [4]. These findings highlight the importance of early identification and accurate risk stratification in patients with suspected sepsis.

The Sepsis-3 criteria define sepsis as a suspected or documented infection accompanied by an acute increase of two or more points in the sequential organ failure assessment (SOFA) score [5]. Although the SOFA scoring system has diagnostic, prognostic, and therapeutic utility in sepsis, it is complex and may lead to delays in sepsis diagnosis [6,7]. Furthermore, among the six organ systems, coagulation and cardiovascular systems could not be reliably categorized into five distinct score groups, raising concerns about the reliability of some SOFA score indices [8]. Although the quick SOFA score (qSOFA) was proposed as a prompt screening tool, several studies have questioned its predictive accuracy and have not recommended its routine use [9,10,11]. Accordingly, there is a growing need to complement clinical scoring systems with objective biomarkers that may facilitate early identification and accurate risk stratification in sepsis.

Procalcitonin (PCT) is a conventional sepsis biomarker that has shown diagnostic, prognostic, and therapeutic utility including antibiotic stewardship [12,13,14,15]. Presepsin (PSEP), a part of the lipopolysaccharide (LPS) receptor, has been reported to be more specific to bacterial infection than PCT [16]. PSEP has shown a diagnostic accuracy comparable to that of PCT, while showing superior prognostic value in predicting sepsis-related mortality [17,18,19,20]. Interferon-λ3 (IFN-λ3), a type III interferon, exhibited delayed kinetics compared to type I interferons; although precise time-to-peak measurements in sepsis are not yet well-defined, this kinetic profile suggests that IFN -λ3 may reflect a later phase of immune response [21,22]. Therefore, when used alongside PCT and PSEP, IFN-λ3 can reflect a broader coverage of sepsis pathophysiology. Several studies reported that increased IFN-λ3 levels are significantly associated with sepsis severity and have prognostic value in predicting sepsis mortality [23,24]. Bioactive adrenomedullin (bio-ADM) is a ubiquitous signaling peptide that plays a crucial role in maintaining vascular integrity and regulating vascular tone in sepsis [25,26,27]. Increased bio-ADM levels were significantly associated with organ dysfunction and higher mortality in sepsis [28,29]. Furthermore, accumulating evidence suggests that multi-marker approaches combining several biomarkers or adding biomarker(s) to the SOFA score would improve diagnostic and prognostic performance compared with using the SOFA score alone [30,31,32,33,34]. A multi-marker approach may complement information reflecting the heterogeneous pathophysiology of sepsis, rather than replacing established clinical scoring systems.

In this study, we aimed to explore the clinical utility (diagnosis, risk stratification, and prognosis) of a multi-marker approach using the four biomarkers (PCT, PSEP, IFN-λ3, and bio-ADM) in sepsis. For this purpose, we assessed whether these biomarkers could differentiate suspected sepsis from confirmed sepsis, stratify sepsis severity, and predict in-hospital mortality. We hypothesized that the multi-marker approach, either alone or in combination with the SOFA score, would enhance clinical utility beyond the SOFA score, especially in patients aged ≥75 years.

## 2. Materials and Methods

### 2.1. Study Population

A total of 527 patients who were admitted to the Konkuk University Medical Center (KUMC) from May 2020 to July 2021 were eligible for study enrollment. They were clinically suspected of having sepsis and had increased CRP levels (>10 mg/dL) in routine testing [35,36]. After excluding 279 patients (due to inappropriate samples [residual sera volume < 1 mL, hemolysis, or clot] and/or age < 20 years old), the remaining 248 patients were finally enrolled in the study. Their medical records were reviewed retrospectively to retrieve demographic, clinical, and laboratory data; the SOFA score was calculated at enrollment. Patients were classified into two groups based on the Sepsis-3 criteria. Suspected sepsis was defined as clinical suspicion of sepsis at the time of blood sampling without fulfillment of the Sepsis-3 criteria during the entire hospitalization. Confirmed sepsis was retrospectively defined according to the Sepsis-3 criteria, requiring a suspected or documented infection [5]. The classification of suspected versus confirmed sepsis was performed after completion of clinical and microbiological assessments and was independent of biomarker results. There was no patient with Coronavirus disease 2019 (COVID-19) in the study population; thus, potential bias was minimized in evaluating sepsis prognosis [37].

Basic characteristics of the study population are summarized in Table 1. The 248 patients were divided into two groups: suspected sepsis (n = 132) and sepsis (n = 116). Septic shock was confirmed in nine patients in the sepsis group; due to the small sample size, further analysis was not conducted in these patients. Accordingly, our analysis focused on the sepsis for which lactate measurements were not essential. A total of 103 patients had microbiologically confirmed infection based on positive culture results. The primary sources of infection were bacteremia (n = 37, 35.9%); respiratory tract (n = 33, 32.0%); urinary tract (n = 16, 15.5%); wound or soft tissue (n = 10, 9.7%); and intra-abdominal sites (n = 7, 6.8%). We performed additional subgroup analysis of biomarker levels according to pathogen type in 103 culture-positive sepsis patients using the Kruskal–Wallis test with post hoc analysis (Dunn). The remaining 13 patients had radiographic findings suggestive of infection. In 45 patients (38.8%), community-acquired sepsis (sepsis onset within ≤72 h of hospital admission without recent exposure to healthcare-associated risk factors) was diagnosed [38]. Comorbidities were assessed using the 19-item version of the Charlson comorbidity index (CCI), which includes myocardial infarction, chronic heart failure, peripheral vascular disease, dementia, chronic pulmonary disease, connective tissue disease, ulcer disease, liver disease, diabetes, hemiplegia, renal disease, malignancy, and acquired immunodeficiency syndrome [39].

This study consisted of a retrospective analysis of medical records and an in vitro measurement of biomarker levels. The study protocol was approved by the institutional review board of KUMC (approval No. 2023-06-057). This retrospective study used anonymized clinical data and involved no additional sampling or intervention; accordingly, the requirement for obtaining written informed consent was waived. This retrospective study was conducted and reported in accordance with the STROBE statement and checklist [40].

### 2.2. Biomarker Assays

Residual plasma and serum samples were collected following routine blood testing. To avoid repeated freeze–thaw cycles, samples were aliquoted and stored at −70 °C until analysis. Prior to biomarker measurements, frozen samples were thawed at room temperature and gently mixed. PCT level was measured in serum using the Elecsys BRAHMS PCT assay (Roche Diagnostics, Basel, Switzerland) based on an electrochemiluminescence immunoassay on a Cobas e801 module (Roche Diagnostics), with a measurement range of 0.02–100 ng/mL [41]. PSEP and IFN-λ3 levels were measured in serum using the HISCL PSEP and HISCL IFN-λ3 assays (Sysmex, Kobe, Japan) based on a chemiluminescence enzyme immunoassay on an HISCL 5000 automated analyzer (Sysmex). The measurement ranges were 20–30,000 pg/mL for PSEP and 3–200 pg/mL for IFN-λ3, respectively; samples with IFN-λ3 levels < 3 pg/mL were diluted, and IFN-λ3 levels were remeasured [42,43,44,45]. Frozen, anonymized plasma samples were sent to a reference laboratory (SphingoTec GmbH, Hennigsdorf, Germany) for bio-ADM level measurement using the sphingotest bio-ADM assay based on an immunoluminometric assay, with a measurement range of 18.8–735 pg/mL [28].

### 2.3. Statistical Analysis

The Shapiro–Wilk test and Grubb’s test were used to check data normality and outliers, respectively. Both SOFA score and biomarker levels were deviated significantly from normality (*p* < 0.001); therefore, non-parametric methods were applied, and continuous variables were summarized using medians and interquartile ranges. No outlier was identified. Distribution of biomarkers and their quartiles (Q1–Q4) were analyzed along with SOFA score using the Mann–Whitney U test, Chi-squared test, and Kruskal–Wallis test with Dunn’s post hoc analysis. The multi-marker approach was categorized into five groups (from 0 to 4) based on the number of biomarkers exceeding their optimal cutoff values. To stratify sepsis severity and prognosis, the SOFA score was also divided into five groups: 0–1; 2–3; 4–5; 6–7; and > 8. Diagnostic and prognostic performances were assessed using the receiver operating characteristic (ROC) curve analysis, with discrimination quantified by the area under the curve (AUC). Optimal cutoff values were determined using Youden’s index, and ROC curves were compared using DeLong’s test. In prognostic analysis, ROC was used solely to determine clinically interpretable cutoff values for individual biomarkers; these cutoffs were subsequently applied in Kaplan–Meier survival analyses and multivariate Cox regression for in-hospital mortality and were independent from diagnostic ROC cutoffs. For the survival analysis, the Kaplan–Meier method was used; continuous variables were dichotomized based on optimal cutoffs, and survival curves were compared using the log-rank test. To address potential limitations of dichotomization, sensitivity analysis was additionally performed using biomarker levels and SOFA score as continuous variables. Biomarker values were natural log-transformed to reduce right skewness, and Cox proportional hazard models were constructed using log-transformed biomarkers. Using the multivariate Cox regression analysis, an adjusted hazard ratio (HR) with a 95% confidence interval (CI) was compared; covariates included in the model were age, sex, CCI, and hypertension. Proportional hazards assumptions were assessed using Schoenfeld residuals for the covariates, and no major violations were observed (all *p* > 0.05). Additionally, Kaplan–Meier curves were visually inspected, which confirmed that the hazards were proportional over time. The sample size for survival analysis was estimated based on a previous study [46]. The inputs were as follows: analysis time t = 7 months; accrual time α = 7 months; follow-up time b = 7 months; null survival probability; S0(t) = 0.244, alternative survival probability of this study; and S1(t) = 0.150. Using arcsine square-root transformation and a two-sided type I error rate (α) of 0.05 and power (1 − β) of 0.8, the estimated sample size was 139, indicating sufficient power for this study. To evaluate the incremental prognostic value beyond the SOFA score, add-on values of biomarkers were estimated using reclassification analysis including integrated discrimination improvement (IDI) and net reclassification improvement (NRI). MedCalc Software (version 22.009, MedCalc Software, Ostend, Belgium), R version 4.3.1, and R Studio 2023.06.0+421 (The R Foundation for Statistical Computing, Vienna, Austria) were used for the statistical analyses. *p* < 0.05 was considered statistically significant.

## 3. Results

The total population consisted of 95 females (38.3%) and 153 males (61.7%), and their median age was 68 years (interquartile range: 57–78 years). The patients aged ≥75 years accounted for 79 patients (31.9%). Age, comorbidities (CCI and hypertension), SOFA score, in-hospital mortality, and the four biomarkers (PCT, PSEP, IFN-λ3, and bio-ADM) differed significantly between the two groups of suspected sepsis and sepsis (Table 1).

**Figure 1:**
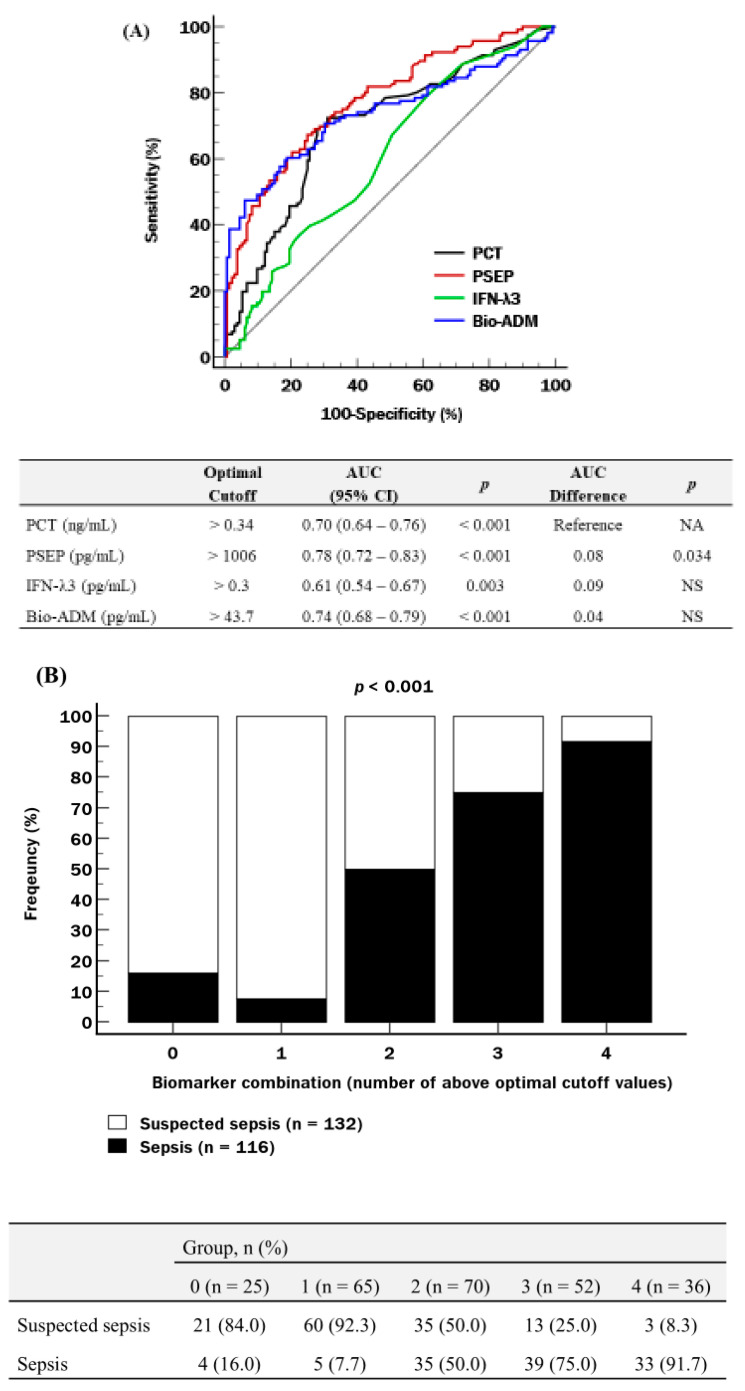
Diagnostic performance of biomarkers for sepsis. (**A**) ROC curves for biomarkers. (**B**) Distribution of sepsis cases stratified by the number of biomarkers exceeding optimal cutoff values. Abbreviations: ROC, receiver operating characteristic; NA, not available; NS, not significant; AUC, area under the curve; CI, confidence interval; PCT, procalcitonin; PSEP, presepsin; IFN-λ3, interferon-λ3; bio-ADM, bioactive adrenomedullin.

All four biomarkers could differentiate suspected sepsis from confirmed sepsis, showing significant diagnostic performance (AUC ranging from 0.61 to 0.78; *p* < 0.001–0.003). PSEP was superior to PCT (*p* = 0.034), and IFN-λ3 and bio-ADM were comparable to PCT (Figure 1A). The proportion of sepsis increased significantly according to the number of biomarkers exceeding optimal cutoff values, showing 91.7% in group 4 (*p* < 0.001) (Figure 1B).

Regarding risk stratification, the distribution of biomarker quartiles differed significantly according to the SOFA score group (all *p* < 0.001). Notably, the proportion of patients in the highest quartile (Q4) increased with higher SOFA scores for each biomarker: PCT (13.9–52.9%); PSEP (7.0–52.9%); IFN-λ3 (10.1–41.2%); and bio-ADM (14.5–57.1%) (Table 2).

**Figure 2:**
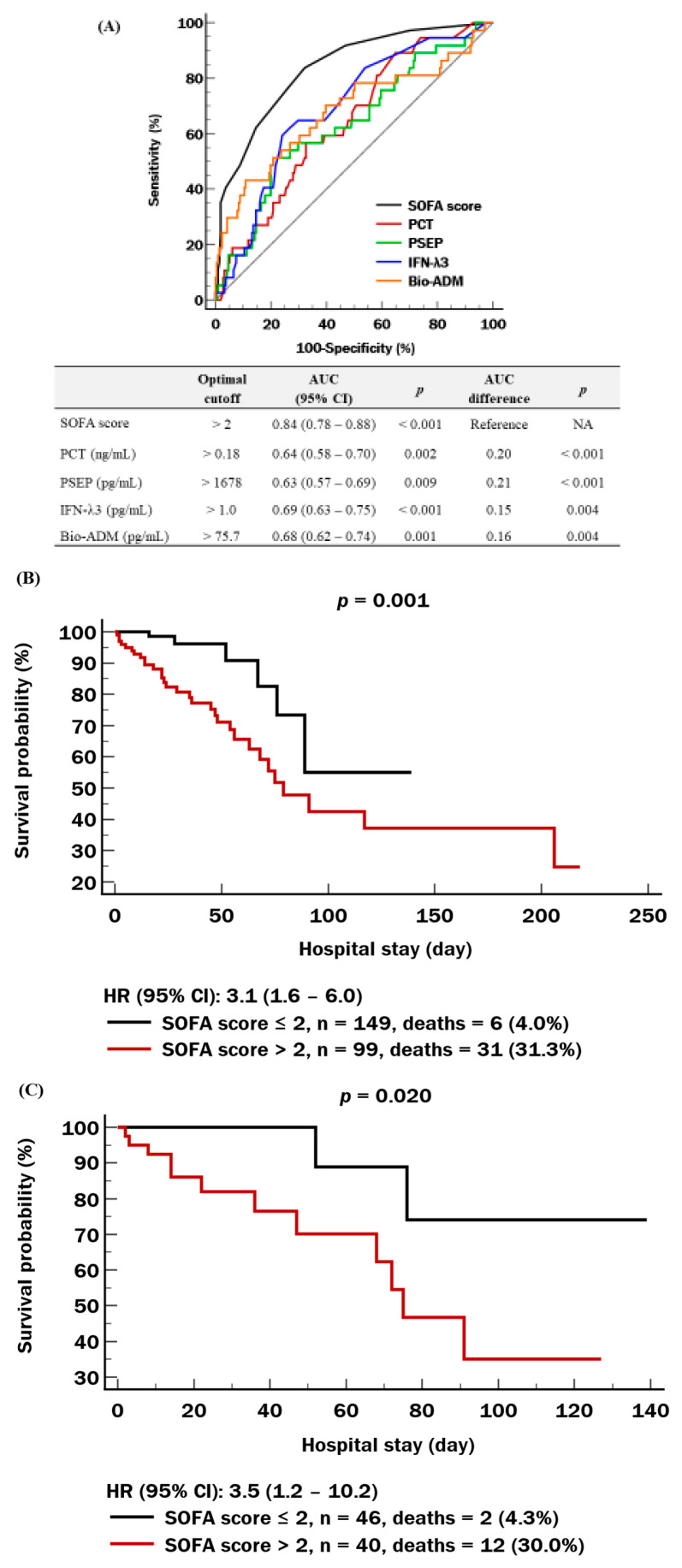

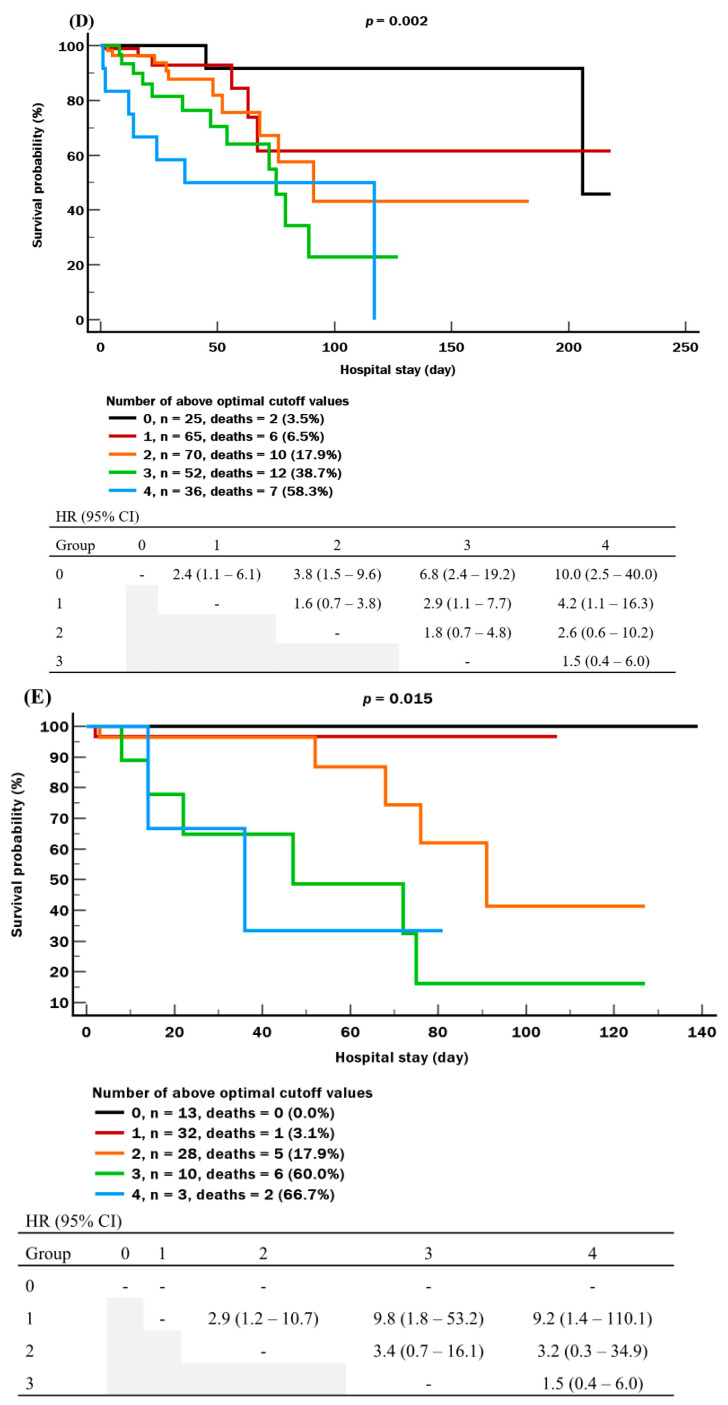
Prognostic performance of the SOFA score and multi-marker approach for in-hospital mortality. (**A**) ROC curves for the SOFA score and biomarkers. (**B**) Survival curves by SOFA score in the overall population. (**C**) Survival curves by SOFA score in patients aged ≥75 years. (**D**) Survival curves by multi-marker approach in the overall population. (**E**) Survival curves by multi-marker approach in patients aged ≥75 years. Colors indicate multi-marker approach groups: black, group 0; red, group 1; orange, group 2; green, group 3; blue, group 4. Abbreviations: SOFA, sequential organ failure assessment score; HR, hazard ratio; see Figure 1.

Regarding prognosis, SOFA score and the four biomarkers predicted in-hospital mortality significantly (AUC ranging from 0.63 to 0.84, *p* < 0.001–0.009), although each biomarker was inferior to the SOFA score (*p* < 0.001–0.004) (Figure 2A). In the total population, the SOFA score showed a mortality rate of 31.3% (HR = 3.1; 95% CI, 1.6–6.0) (Figure 2B). In the patients aged ≥75 years, the SOFA score showed a similar performance (mortality rate of 30.0%; HR = 3.5; 95% CI, 1.2–10.2) (Figure 2C). In the total population, the multi-marker approach showed a stepwise increase in the mortality rate across groups (ranging from 3.5% to 58.3%). The HR of group 4, compared with group 1, was 4.2 (95% CI, 1.1–16.3) (Figure 2D). In the patients aged ≥75 years, a similar finding was observed, with mortality rates increasing from 0% in group 0 to 66.7% in group 4, yielding an HR of 9.2 (95% CI, 1.4–110.1) (Figure 2E).

In the multivariate Cox regression analysis for predicting in-hospital mortality, the adjusted HR for the SOFA score was 4.6 (95% CI, 1.9–11.4) (Table 3). For the biomarkers, adjusted HRs (95% CI) were as follows: PCT, 5.3 (1.6–18.0); PSEP, 1.5 (1.1–3.1); IFN-λ3, 2.2 (1.2–4.4); and bio-ADM, 4.0 (2.1–7.9). When the biomarkers were combined, there was a stepwise increase in adjusted HRs across groups, ranging from 2.6 to 14.7 compared with group 0. Compared with the SOFA score, group 4 showed more than a threefold increased risk of predicting in-hospital mortality. In sensitivity analysis using log-transformed continuous biomarkers, the SOFA score remained independently associated with mortality (HR 1.28; 95% CI 1.14–1.43; *p* < 0.0001). Among the biomarkers, PSEP (HR 0.62; 95% CI 0.41–0.94; *p* = 0.024) and bio-ADM (HR 1.77; 95% CI 1.05–2.97; *p* = 0.032) were independently associated with mortality, whereas PCT (HR 1.11; 95% CI 0.89–1.39; *p* = 0.336) and IFN-λ3 (HR 1.23; 95% CI 0.89–1.69; *p* = 0.204) were not statistically significant.

In the reclassification analysis, when a single biomarker was added to the SOFA score, all the combinations showed no statistical significance (Table 4). Adding two biomarkers yielded significant improvement in two out of the six combinations, with IDI values ranging from 0.37 to 0.41 (*p* = 0.05) and NRI values from 0.74 to 0.75 (*p* = 0.04–0.05). When three biomarkers were added, three out of the four combinations showed significant improvement, with IDI values from 0.38 to 0.44 (*p* = 0.01–0.05) and NRI values from 0.75 to 0.82 (*p* = 0.03–0.05). When all four biomarkers were added to the SOFA score, the combination consistently showed significant improvement, with an IDI value of 0.45 (*p* = 0.03) and an NRI value of 0.79 (*p* = 0.02).

## 4. Discussion

This is the first study that evaluated the clinical utility of four sepsis-related biomarkers (PCT, PSEP, IFN-λ3, and bio-ADM) comprehensively in a cohort of patients with suspected sepsis. Although these biomarkers have been investigated individually, their combined use regarding clinical utility, such as diagnosis, risk stratification, and prognosis prediction, remains underexplored. Our results suggest that a multi-marker approach may provide incremental or complementary clinical utility on top of the SOFA score.

In the diagnosis of sepsis, PCT and PSEP showed the optimal cutoff values of 0.34 ng/mL and 1006 pg/mL, respectively (Figure 1A). As a conventional biomarker, PCT thresholds are well-established in clinical practice (>0.5–2 ng/mL to rule in sepsis; <0.25–0.55 ng/mL to rule out sepsis) [47]. PSEP showed variable cutoff values across studies, with reported ranges of 207–907 pg/mL in adults and 299–1014 in pediatrics [48]. In this study, PSEP showed superior diagnostic performance to PCT, with a significantly higher AUC. These results are consistent with previous findings that PSEP showed comparable or superior diagnostic performance compared with PCT [18,19,20]. Furthermore, PSEP has a time advantage in the early detection of sepsis, with a response time of less than two hours, compared with two to three hours for PCT [16]. These findings imply that PSEP may be more effective than PCT for early sepsis diagnosis, which could facilitate earlier clinical decision-making and thereby improve prognosis. To our knowledge, there are no established diagnostic cutoff values for IFN-λ3 or bio-ADM in sepsis. In this study, the preliminary thresholds were identified as 0.3 pg/mL for IFN-λ3 and 43.7 pg/mL for bio-ADM (Figure 1A). Although the IFN-λ3 threshold falls below the assay’s reported lower limit of 3 pg/mL, all samples below the lower limit were diluted and remeasured according to the assay protocol, enabling reliable quantification. These thresholds should be interpreted with caution and are not definitive diagnostic criteria. Although these thresholds require further validation in larger, multicenter cohorts before clinical adoption, both IFN-λ3 and bio-ADM showed diagnostic performance comparable to PCT. These findings suggest their potential utility as alternative or complementary tools for sepsis diagnosis.

Sepsis is a complex, dysregulated host response to infection, involving multifaceted immunologic, hemodynamic, and metabolic disturbances rather than a simple infectious disease [17]. Given this complexity, it would be difficult to reflect the full spectrum of sepsis pathophysiology with a single biomarker. Consequently, using multiple biomarkers reflecting different biological pathways has emerged as a promising strategy to enhance clinical performance. Building on this rationale, this study evaluated a multi-marker approach combining four biomarkers representing distinct pathophysiological pathways. In this study, the proportion of sepsis increased to 91.7% when all four biomarkers exceeded their optimal cutoff values (Figure 1B). On the contrary, when only one biomarker increased among the four biomarkers, the sepsis proportion was only 7.7%. These results suggest that a multi-marker approach may provide significantly better diagnostic accuracy for sepsis compared with relying on a single biomarker.

In the context of risk stratification, the distribution of each biomarker across the SOFA score group showed a clear trend according to sepsis severity, suggesting a potential role in reflecting the degree of organ dysfunction (Table 2). Notably, only bio-ADM showed a significant association with the SOFA cardiovascular subscore (*p* = 0.049). This finding supports the role of bio-ADM as a surrogate marker for mean arterial pressure within the SOFA score. Given its role in regulating vascular tone and maintaining endothelial integrity during sepsis [25,26,27], increased bio-ADM levels may reflect sepsis-induced vasodilation and hemodynamic instability [28,29]. Its association with the SOFA cardiovascular subscore further highlights its potential as a clinically relevant marker of circulatory dysfunction in septic patients.

Although each individual biomarker showed prognostic value for in-hospital mortality, their prognostic performance was lower than that of the SOFA score (Figure 2). For the multi-marker approach, all possible combinations of the SOFA score with the four biomarkers were evaluated to demonstrate incremental prognostic values [30,31,32,33,34]. Notably, patients with elevations in all four biomarkers (group 4) showed a markedly higher mortality rate (58.3%) compared with those with elevated SOFA scores (31.3%) in the total population. The difference was even more pronounced in patients aged ≥75 years, where the mortality rate in group 4 rose to 66.7%, while it decreased to 30.0% using the SOFA score. Similarly, the HR of group 4 rose from 4.2 in the total population to 9.2 in patients aged ≥75 years, whereas the HR associated with the SOFA score had similar values (3.1 vs. 3.5, respectively). Furthermore, in patients aged ≥75 years, group 0 of the biomarker combination (no elevation in any of the biomarkers) showed a mortality rate of 0% (Figure 2E). These results suggest that the multi-marker approach may not only identify high-risk patients more accurately than the SOFA score but also effectively delineate those with minimal mortality risk. But given the small sample sizes in patients aged ≥75 years, these findings should be interpreted cautiously. The multi-marker approach may provide complementary information for identifying high- and low-risk patients, without implying superiority over the SOFA score. Group 2 of biomarker combination showed an adjusted HR comparable to that of the SOFA score when using group 0 as a reference (4.5 vs. 4.6), while group 4 exhibited a nearly threefold higher adjusted HR (14.7 vs. 4.6) (Table 3). These results underscore the independent predictive value of the multi-marker approach beyond the SOFA score; this is in line with unadjusted mortality data, showing higher mortality rates and HRs in the biomarker combination than in the SOFA score. Furthermore, reclassification analysis (IDI and NRI) showed that adding multiple biomarkers on top of the SOFA score improved the prognostic performance up to 82% (Table 4). These findings support the conceptual basis of the multi-marker approach, which aims to reflect the complex and heterogeneous pathophysiology of sepsis. Sensitivity analysis using continuous, log-transformed biomarkers in Cox regression showed that only PSEP and bio-ADM retained independent associations with in-hospital mortality. However, Cox regression, Kaplan–Meier curves, and ROC analyses using clinically interpretable cutoffs consistently demonstrated prognostic performance for all four biomarkers, supporting the validity of dichotomization for practical mortality risk assessment.

In additional subgroup analysis, PCT was significantly higher in Gram-negative bacterial infections (median 1.2 ng/mL) compared with Gram-positive infections (0.5 ng/mL); IFN-λ3 was significantly higher in fungal infections (4.6 pg/mL) compared with Gram-positive or Gram-negative infections (0.5 and 0.7 pg/mL, respectively), and no significant differences were observed in PSEP and bio-ADM. These findings suggest a trend consistent with previous observations that PCT levels tend to be higher in bacterial infections [49]. Further validation in larger, multicenter cohorts is needed before clinical adoption.

This study has several limitations. First, 279 samples were excluded primarily due to inadequate residual serum volume or age < 20 years. This exclusion may introduce selection bias with a study population disproportionately composed of elderly individuals (median age: 68 years). Accordingly, our findings may not be generalizable to the entire age spectrum including pediatric patients. Given that sepsis remains a leading cause of death in children, further studies are needed to evaluate the clinical utility of these biomarkers in the pediatric population [50]. Second, the SOFA score was assessed only at the time of patient enrollment without information on the patients’ baseline SOFA scores. In accordance with the Sepsis-3 criteria, the SOFA score of zero was assumed in patients without known organ dysfunction [5]. This approach, however, may limit our ability to accurately calculate acute increases in the SOFA score, particularly in patients with chronic comorbidities or pre-existing organ dysfunction. Therefore, potential misclassification of sepsis status cannot be completely ruled out. Third, this study was primarily designed to assess the diagnostic and prognostic utility of biomarkers. Considering the importance of antimicrobial stewardship and reducing unnecessary antibiotic use, future studies should also explore the therapeutic implications of biomarker-guided interventions. Fourth, this study compared the biomarkers with only the SOFA score. Hematologic inflammation indices based on complete blood count could provide advantages over the SOFA score, as they are easily obtainable, require no clinical judgment, and can be calculated quickly. These indices include the neutrophil-to-lymphocyte ratio, platelet-to-lymphocyte ratio, lymphocyte-to-monocyte ratio, systemic immune inflammation index, and systemic inflammation response index [51]. Accordingly, future studies will be needed to evaluate the clinical utility of these hematologic indices for improving the assessment and management of sepsis. Fifth, conventional inflammatory markers such as erythrocyte sedimentation rate were not assessed in this study. Although CRP levels were measured, they were used solely for sample selection and not for diagnosis or prognostic evaluation. These markers should also be comprehensively evaluated in future studies.

## 5. Conclusions

In conclusion, biomarkers and their combination showed clinical utility in patients with suspected sepsis. The multi-marker approach showed complementary clinical utility in addition to the SOFA score and showed better prognostic performance in patients aged ≥75 years. A biomarker, alone or in combination, would be a valuable tool added on top of the SOFA score. Further studies are awaited to validate the clinical utility of the multi-marker approach and establish its role in routine sepsis management.

## Figures and Tables

**Table 1:** Basic characteristics of the study population.

	Total (n = 248)	Suspected Sepsis (n = 132)	Sepsis (n = 116)	*p*
**Demographics**				
Age (yr)	68 (57–78)	64 (51–75)	73 (63–82)	<0.001
Aged 75 or more, n (%)	79 (31.9)	31 (23.5)	48 (41.4)	0.003
Female, n (%)	95 (38.3)	79 (59.8)	74 (63.8)	NS
**Comorbidities**				
CCI	5 (3–6)	4 (2–5)	6 (5–7)	<0.001
Hypertension, n (%)	104 (41.9)	46 (34.8)	58 (50)	0.016
**Patient enrollment**				
General ward, n (%)	210 (84.7)	118 (89.4)	92 (79.3)	0.028
Emergency room, n (%)	21 (8.5)	11 (8.3)	10 (8.6)	NS
ICU, n (%)	17 (6.9)	3 (2.3)	14 (12.1)	0.024
**SOFA score at enrollment**				
Total	2 (0–4)	1 (0–1)	4 (3–6)	<0.001
Central nervous system	0 (0–1)	0 (0–0)	0 (0–2)	<0.001
Renal	0 (0–1)	0 (0–1)	0 (0–2)	<0.001
Respiratory	0 (0–1)	0 (0–1)	1 (0–1)	<0.001
Coagulation	0 (0–0)	0 (0–0)	0 (0–1)	<0.001
Circulatory	0 (0–0)	0 (0–0)	0 (0–0)	<0.001
Liver	0 (0–0)	0 (0–0)	0 (0–2)	<0.001
**Clinical outcomes**				
Hospital stay (day)	17 (9–44)	12 (7–26)	29 (13–53)	<0.001
ICU stay (day)	3 (2–12)	2 (1–4)	5 (2–22)	0.006
ICU admission, n (%)	73 (29.4)	26 (19.7)	47 (40.5)	<0.001
In-hospital mortality, n (%)	37 (15.0)	6 (4.6)	31 (26.7)	<0.001
**Laboratory data at enrollment**				
WBC (×10^9^/L)	10.6 (7.9–14.4)	10.2 (7.4–14.8)	11.0 (8.6–14.9)	NS
Hemoglobin (g/dL)	10.1 (9.0–11.8)	10.5 (9.5–12.7)	9.7 (8.6–11.0)	<0.001
Platelets (×10^9^/L)	223 (152–291)	245 (190–319)	184 (122–266)	<0.001
Lactate (mmol/L) *	1.7 (1.3–2.5)	1.6 (1.2–2.0)	1.7 (1.3–2.8)	0.012
Total bilirubin (umol/L)	0.7 (0.5–1.1)	0.6 (0.4–0.9)	0.7 (0.5–1.9)	<0.001
Creatinine (μmol/L)	0.9 (0.6–1.3)	0.8 (0.6–1.0)	1.2 (0.7–2.2)	<0.001
CRP (mg/dL)	15.9 (12.6–21.0)	15.7 (12.4–21.5)	15.5 (12.8–20.8)	NS
PCT (ng/mL)	0.35 (0.15–1.50)	0.20 (0.11–0.57)	0.65 (0.25–2.54)	<0.001
PSEP (pg/mL)	879 (496–1696)	589 (388–1011)	1494 (800–2748)	<0.001
IFN-λ3 (pg/mL)	0.5 (0.3–1.4)	0.5 (0.2–1.0)	0.6 (0.4–1.8)	0.004
Bio-ADM (pg/mL)	36.6 (21.7–59.9)	28.6 (19.1–40.9)	56.1 (30.3–88.0)	<0.001

Data are represented as number (percentage) or median (interquartile range). * Lactate level was measured in 176 patients (suspected sepsis, n = 75; sepsis, n = 101), and septic shock was confirmed in nine patients. Abbreviations: CCI, Charson comorbidity index; NS, not significant; ICU, intensive care unit; SOFA, sequential organ failure assessment; WBC, white blood cells; CRP, C-reactive protein; PCT, procalcitonin; PSEP, presepsin; IFN-λ3, interferon-λ3; bio-ADM, bioactive adrenomedullin.

**Table 2:** Biomarker quartile distribution by SOFA score groups.

	SOFA Score, n (%)					*p*
	0–1 (n = 115)	2–3 (n = 51)	4–5 (n = 45)	6–7 (n = 20)	>8 (n = 17)	
**PCT**						<0.001
Q1	34 (29.6)	15 (29.4)	8 (17.8)	4 (20.0)	1 (5.9)	
Q2	46 (40.0)	5 (9.8)	6 (13.3)	2 (10.0)	2 (11.8)	
Q3	19 (16.5)	18 (35.3)	16 (35.6)	5 (25.0)	5 (29.4)	
Q4	16 (13.9)	13 (25.5)	15 (33.3)	9 (45.0)	9 (52.9)	
**PSEP**						<0.001
Q1	49 (42.6)	10 (19.6)	1 (2.2)	0 (0.0)	2 (11.8)	
Q2	32 (27.8)	15 (29.4)	8 (17.8)	4 (20.0)	3 (17.6)	
Q3	26 (22.6)	13 (25.5)	14 (31.1)	6 (30.0)	3 (17.6)	
Q4	8 (7.0)	13 (25.5)	22 (48.9)	10 (50.0)	9 (52.9)	
**IFN-λ3**						<0.001
Q1	30 (26.1)	8 (15.7)	10 (22.2)	1 (5.0)	1 (5.9)	
Q2	27 (23.5)	12 (23.5)	6 (13.3)	4 (20.0)	4 (23.5)	
Q3	36 (31.3)	16 (31.4)	11 (24.4)	13 (65.0)	5 (29.4)	
Q4	22 (19.1)	15 (29.4)	18 (40.0)	2 (10.0)	7 (41.2)	
**Bio-ADM**						<0.001
Q1	39 (29.8)	16 (25.8)	7 (14.6)	4 (17.4)	1 (4.8)	
Q2	49 (37.4)	10 (16.1)	8 (16.7)	2 (8.7)	4 (19.0)	
Q3	24 (18.3)	20 (32.3)	17 (35.4)	8 (34.8)	4 (19.0)	
Q4	19 (14.5)	16 (25.8)	16 (33.3)	9 (39.1)	12 (57.1)	

Abbreviations: Q, quartile; see Table 1.

**Table 3:** Cox regression analysis of SOFA score and biomarkers for predicting in-hospital mortality.

	Number at Risk	Number of Events	Crude HR	Adjusted HR * (95% CI)
**SOFA score > 2**				
No	149	6	1.0	1.0 (reference)
Yes	99	31	3.9	4.6 (1.9–11.4)
**PCT > 0.18 ng/mL**				
No	78	4	1.0	1.0 (reference)
Yes	170	33	5.1	5.3 (1.6–18.0)
**PSEP > 1678 pg/mL**				
No	185	18	1.0	1.0 (reference)
Yes	63	19	1.5	1.5 (1.1–3.1)
**IFN-λ3 > 1.0 pg/mL**				
No	175	15	1.0	1.0 (reference)
Yes	73	22	2.3	2.2 (1.2–4.4)
**Bio-ADM > 75.7 pg/mL**				
No	209	21	1.0	1.0 (reference)
Yes	39	16	3.9	4.0 (2.1–7.9)
**Multi-marker approach** **grouped by number of optimal cutoff values**				
Group 0	57	2	1.0	1.0 (reference)
Group 1	92	6	2.6	2.6 (0.5–13.4)
Group 2	56	10	4.5	4.5 (1.3–22.3)
Group 3	31	12	8.3	8.5 (1.7–43.0)
Group 4	12	7	12.1	14.7 (2.7–80.3)

* Covariates used for adjusted HR were age, sex, Charlson comorbidity index, and hypertension. Abbreviations: HR, hazard ratio; CI, confidence interval; see Table 1.

**Table 4:** Reclassification analysis of SOFA score and biomarkers for predicting in-hospital mortality.

	IDI (95% CI)	*p*	NRI (95% CI)	*p*
**SOFA score alone**				
SOFA	Reference	NA	Reference	NA
**SOFA score with one biomarker**				
SOFA + PCT	-	NS	-	NS
SOFA + PSEP	-	NS	-	NS
SOFA + IFN	-	NS	-	NS
SOFA + bio-ADM	-	NS	-	NS
**SOFA score with two biomarkers**				
SOFA + PCT + PSEP	0.37 (0.01–0.67)	0.050	0.75 (0.01–1.35)	0.050
SOFA + PCT + IFN	0.41 (0.01–1.04)	0.050	0.74 (0.01–1.94)	0.040
SOFA + PCT + bio-ADM	-	NS	-	NS
SOFA + PSEP + IFN	-	NS	-	NS
SOFA + PSEP + bio-ADM	-	NS	-	NS
SOFA + IFN + bio-ADM	-	NS	-	NS
**SOFA score with three biomarkers**				
SOFA + PCT + PSEP + IFN	0.44 (0.01–0.82)	0.047	0.82 (0.01–1.49)	0.047
SOFA + PCT + PSEP + bio-ADM	0.38 (0.11–1.31)	0.010	0.75 (0.01–2.50)	0.050
SOFA + PCT + IFN + bio-ADM	0.42 (0.01–0.92)	0.050	0.79 (0.01–1.61)	0.030
SOFA + PSEP + IFN + bio-ADM	-	-	-	NS
**SOFA score with four biomarkers**				
SOFA + PCT + PSEP + IFN + bio-ADM	0.45 (0.01–1.00)	0.030	0.79 (0.01–1.34)	0.020

The number of iterations for the perturbation resampling was 200. Only the values with statistical significance are presented. Abbreviations: NA, not available; NS, not significant; SOFA, sequential organ failure assessment; PCT, procalcitonin; PSEP, presepsin; IFN, interferon-λ3; bio-ADM, bioactive adrenomedullin.

## Data Availability

The raw data supporting the conclusions of this article will be made available by the authors upon reasonable request.
